# Social Inequalities in Tooth Loss Over Time: Insights From Australian Adults

**DOI:** 10.1016/j.identj.2025.103860

**Published:** 2025-08-23

**Authors:** Xiangqun Ju, Liana Luzzi, Sergio Chrisopoulos, Gloria C. Mejia, Lisa M. Jamieson

**Affiliations:** Australian Research Centre for Population Oral Health, Adelaide Dental School, The University of Adelaide, Adelaide, South Australia, Australia

**Keywords:** Social inequality, Edentulism, Non-functional dentition, Equivalised household income, Income-related inequality

## Abstract

**Introduction and aim:**

In contemporary society, social inequality in tooth loss is a significant and often overlooked issue. This study aimed to describe and examine social differentials in tooth loss among Australian adults over time.

**Methods:**

This analysis used data from the National Survey of Adult Oral Health (NSAOH), which was conducted in Australia in 2 waves: NSAOH-1 (2004-2006) and NSAOH-2 (2017-2018). The study employed a population-based cross-sectional design, with adults aged 15 years and older randomly selected using a 3-stage, stratified sampling method across metropolitan and regional areas in each state and territory. The primary outcomes were self-reported edentulism and non-functional dentition (<21 teeth). Explanatory variables included socioeconomic status, assessed using equivalized household income and grouped into approximate quartiles from lowest to highest, along with sociodemographic factors, CALD (Culturally and Linguistically Diverse) status, and oral health-related behaviour. Unadjusted and adjusted absolute prevalence differences (PDs) were calculated to assess income-related inequality. Adjustments were made for age and sex using the average covariate distribution across all income groups.

**Results:**

Data were available for 14,123 participants in NSAOH-1 and 15,731 participants in NSAOH-2. The prevalence of edentulism declined from 6.4% to 4.0%, and non-functional dentition decreased from 17.1% to 13.8% over time. These reductions were observed across all household income quartiles, with lower prevalence rates for both conditions in NSAOH-2 compared to NSAOH-1. The adjusted model showed that the prevalence decreases were most marked for the lowest household income group, from 15.3 to 6.5 (PD = 8.8, 95% CI: 8.3-9.4) for edentulism and from 35.0 to 21.3 (PD = 13.7, 95% CI: 13.4-14.0) for non-functional dentition.

**Conclusion:**

Our study indicated social inequalities in both edentulism and non-functional dentition among Australian adults over time. Age, sex, place of residence, irregular dental visits, and lack of dental insurance were important factors associated with tooth loss.

## Introduction

In contemporary society, social inequality in tooth loss is a significant and often overlooked issue. It represents a complex interplay between socioeconomic status, oral health and related behaviours, and utilization of oral health services.^1^ Socioeconomic inequalities are frequently observed in dental diseases, such as dental caries^2^ and periodontal disease.^3^

Tooth loss is the ultimate endpoint of these oral diseases. The estimated global average prevalence of edentulism is almost 7% among people aged 20 years or older^4^ and was 4% among people aged 15 or over in Australia.^5^ Recent research indicates that the global age-standardized disability-adjusted life years (DALYs) rate for edentulism declined by 9.08% between 1990 and 2021.^6^

Tooth loss is a key indicator of oral health status. It impairs masticatory function, reduces chewing efficiency, and limits nutrient intake, thereby affecting both oral and general health-related quality of life. Additionally, compromised nutrition and oral function can weaken the immune system, contributing to the development of systemic diseases and potentially increasing the risk of mortality.^7^^,^^8^ The World Health Organization (WHO) has set a global target to achieve a 10% relative reduction in the combined prevalence of major oral diseases, including tooth loss (such as edentulism and having 20 or fewer natural teeth), across the life course by 2030.^9^

Studies have consistently shown that tooth loss is more prevalent among individuals with lower socioeconomic status compared to those with higher socioeconomic standing.^10^, ^11^ Household income is 1 of the primary factors contributing to this inequality. In addition, irregular dental visits play a crucial role in the social inequality of tooth loss.^12^ Regular dental check-ups and preventive care are essential for maintaining oral health and preventing tooth loss.^13^

Thus, the study aimed to describe and examine social differentials in tooth loss among Australian adults over time.

## Methods

This study follows the STROBE (Strengthening the Reporting of Observational Studies in Epidemiology) guidelines for transparent and comprehensive reporting.

### Study design and sample selection

Population-based cross-sectional study data for the present analysis came from the latest availability of comparable national data: National Survey of Adult Oral Health (NSAOH) in 2004-06 (NSAOH-1) and 2017-18 (NSAOH-2) in Australia. Adults aged 15 years and over were randomly selected through a 3-stage, stratified sample design within metropolitan and regional areas in each state/territory. Detailed information on the methods is found in other publications^14^^,^^15^ Data were weighted following standard procedures for clustered samples. Individuals who were selected were invited to respond to a questionnaire. The weighting process is described in detail elsewhere.^14^^,^^15^ Briefly, each participant’s initial weight was defined as the inverse of their survey selection probability. To account for variation in response rates across postcodes, these weights were adjusted to assign greater weight to areas with lower participation. In the next stage, the weights were further calibrated to ensure that the socioeconomic composition of the weighted interview sample accurately reflected the Australian population aged 15 years and over. The socioeconomic variables used in the weighting procedure included age, sex, country of birth, Indigenous status, educational attainment, employment status, housing tenure, and household size.

### Data collection

Self-reported oral health and related characteristics were obtained through computer-assisted telephone interviews (CATI) during 2004-2006, and through either CATI or an online questionnaire in 2017-2018.

### Variables

#### Outcome variables

Outcome variables were self-reported edentulism and non-functional dentition (<21 teeth). Self-reported edentulous status was from dentate status, which was dichotomized into “Dentate” and “Edentulous.” The self-reported number of teeth was classified as “nonfunctional dentition (<21 teeth, including edentulism)” or “functional dentition (≥ 21 teeth).”

#### Explanatory variables

Explanatory variables included socioeconomic status, sociodemographic characteristics, culturally and linguistically diverse (CALD) status, and oral health-related behaviours were collected from interview questionnaires:

1) Social-economic status was estimated by using the equivalized household income, which was derived by calculating an equivalence factor according to the modified OECD (the Organization for Economic Cooperation and Development) equivalence scale,^14^ then dividing income by the equivalence factor which was built up by allocating points to each person in a household (1 point to the first adult, 0.5 points to each additional person who was 15 years and over, and 0.3 to each child under the age of 15 years), then summing the equivalence points of all household member. Inflation-adjusted equivalized household income was calculated for NSAOH-1, with AU$100 in 2004 considered equivalent to AU$138 in 2017.^16^ Household incomes were then grouped into approximate quartiles, from lowest to highest. In NSAOH-1 (2004-06), the quartiles were: <AU$26,932**,** AU$26,932 to <AU$46,000**,** AU$46,000 to <AU$73,600**,** and AU$73,600+. For NSAOH-2 (2017-18), the corresponding quartiles were: **<**AU$28,571**,** AU$28,571 to <AU$44,827**,** AU$44,827 to <AU$73,912**,** and AU$73,912+**.**
2) Sociodemographic characteristics included age (“15-34,” “35-54” or “55 or over” years), sex (“Male” vs “Female”) and residential location (“Major city” vs “Regional/Remote”).
3) CALD status was from 2 questions 1) “What language do you mainly speak at home?” and included 10 response options (English, Northern European – excluding English, Southern European, Eastern European, Southwest and Central Asian, Southern Asian, Southeast Asian, Eastern Asian, Australian Indigenous, Other language) and 2) “In which country were you born?,” with options grouped into 10 response categories (Australia, New Zealand, rest of Oceania, UK and Ireland, rest of Europe, North Africa and Middle East, Asia, USA and Canada, rest of Americas, Sub-Saharan Africa), then was categorized as ‘CALD’ (born overseas and not speaking English as the primary language at home), or ‘All others’.
4) Oral health-related behaviours included last dental visit and dental insurance status. The last dental visit was from the question “How long ago did you last see a dental professional about your teeth, dentures or gums?” and dichotomized options into <12 months and ≥12 months or never visited. Dental insurance was derived from 3 progressive questions: “Do you have a private health insurance other than Medicare?,” “What type of private medical insurance do you have?” and “Does your private health insurance provide cover dental services?.” People who responded “yes” were categorized as having private dental insurance. Otherwise, there was “No.” In the Australian health insurance system, dental care is generally not covered under the public health scheme, Medicare**.** Most dental services must be paid for out-of-pocket or through private health insurance**,** except for limited publicly funded dental programs available to specific eligible groups, such as children or concession card holders.^16^

### Statistical analysis

Basic descriptive analyses were performed to determine the percentage distribution of sample characteristics, the prevalence of edentulism and non-functional dentition, and their corresponding 95% confidence intervals (CIs). Unadjusted and adjusted absolute prevalence differences (PDs) with 95% CIs were estimated to assess household income inequality. Adjustments for age and sex were applied based on the average covariate distribution across the 4 household income groups. Non-overlapping 95% confidence intervals were used as a conservative indication of statistical significance, and the Cochran-Armitage test for trend was considered significant at a p-value < .05. Additionally, multivariable logistic regression models were employed to estimate associations between tooth loss (edentulism and non-functional dentition) and explanatory variables. Odds ratios (ORs) with corresponding 95% confidence intervals (CIs) were reported from the multivariable analyses.

Sensitivity analysis was performed using imputed data. Missing values were addressed through Multiple Imputation (MI) under the assumption that data were missing at random (MAR). The Fully Conditional Specification (FCS) method was applied, utilizing logistic regression for categorical variables and linear regression for continuous variables. All missing data, including both outcome and explanatory variables, were imputed.

Data management and computation of summary variables were performed using SAS software, version 9.4 (SAS Institute Inc., Cary, NC, USA). Sampling weights were applied to account for the survey's complex sampling design.

## Results

Data were available for 14,123 participants in NSAOH-1 and 15,731 participants in NSAOH-2, respectively. The sample characteristics and prevalence with edentulism and non-functional dentition are shown in Table 1. Between the 2 survey periods, statistically significant increases were observed among individuals identifying as CALD (9.7%-14.4%), those in the lowest household income quartile (20.3%-30.4%), and individuals whose last dental visit was more than 12 months ago (40.6%-43.6%).

**Table 1:** Sample characteristics and changes in % edentulism, % nonfunction dentation among Australian adults across time, NSAOH 2004-06 and NSAOH 2017-18, weighted estimates.

	NSAOH 2004-06 (N = 14,123)			NSAOH 20017-18 (N = 15,731)		
	Total sample	Edentulism	Non-functional dentition	Total sample	Edentulism	Non-functional dentition
	% (95% CI)	% (95% CI)	% (95% CI)	% (95% CI)	% (95% CI)	% (95% CI)
Total	**100**	6.4 (6.0-6.9)	17.1 (16.2-18.0)	100	4.0 (3.6-4.4)	13.8 (13.0-14.6)
Age group (years)						
15-34	34.8 (33.5-36.1)	0.0 (0.0-0.0)	0.4 (0.2-0.6)	34.5 (33.4-35.7)	0.0 (0.0-0.0)	0.7 (0.3-1.1)
35-54	35.5 (34.4-36.6)	1.7 (1.3-2.1)	8.4 (7.4-9.4)	32.6 (31.5-33.6)	1.1 (0.7-1.5)	5.9 (5.0-6.8)
55+	29.7 (28.5-30.9)	19.6 (18.2-20.9)	47.1 (45.0-49.1)	32.9 (32.0-33.8)	11.1 (10.1-12.2)	35.5 (33.7-37.3)
Sex						
Male	49.4 (48.3-50.5)	5.2 (4.6-5.7)	15.2 (14.1-16.3)	49.2 (48.1-50.4)	3.4 (2.8-3.7)	13.1 (12.1-14.1)
Female	50.6 (49.5-51.7)	7.6 (7.0-8.3)	18.9 (17.8-20.1)	50.8 (49.6-51.9)	4.7 (4.1-5.2)	14.5 (13.4-15.5)
Residential location						
Regional/remote	33.1 (29.8-36.3)	9.2 (8.3-10.1)	21.9 (20.3-23.5)	28.2 (25.1-31.3)	5.4 (4.7-6.1)	18.2 (16.7-19.8)
Major city	66.9 (63.7-70.2)	5.1 (4.6-5.6)	14.7 (13.7-15.8)	71.8 (68.7-74.9)	3.5 (3.0-3.9)	12.1 (11.1-13.0)
CALD status*						
CALD	9.7 (8.7-10.8)	3.9 (2.8-4.9)	14.2 (11.5-16.8)	14.4 (13.0-15.9)	3.9 (2.6-5.3)	13.7 (11.3-16.1)
All others	90.3 (89.2-91.3)	6.7 (6.2-7.2)	17.4 (16.5-18.3)	85.6 (84.1-87.0)	4.0 (3.7-4.4)	13.8 (13.0-14.6)
Equivalised household income						
Quartile 1 (low)	20.3 (19.2-21.4)	17.1 (15.4-18.7)	38.6 (36.2-41.0)	30.4 (28.9-31.8)	7.2 (6.1-8.4)	23.6 (21.5-25.7)
Quartile 2	28.2 (27.1-29.2)	8.7 (7.7-9.7)	23.1 (21.5-24.6)	23.8 (22.7-24.8)	5.8 (5.0-6.6)	20.1 (18.3-21.8)
Quartile 3	29.2 (28.0-30.4)	1.8 (1.4-2.2)	7.1 (6.2-8.1)	25.2 (24.1-26.3)	1.5 (0.9-2.1)	6.5 (5.4-7.5)
Quartile 4 (high)	22.3 (21.1-23.5)	1.4 (1.0-1.8)	6.3 (5.3-7.3)	20.7 (19.5-21.9)	0.7 (0.3-1.0)	4.2 (3.3-5.1)
Last dental visit						
12+ months ago	40.6 (39.5-41.8)	12.7 (11.7-13.6)	24.3 (22.9-25.7)	43.6 (42.4-44.9)	7.1 (6.4-7.9)	17.8 (16.5-19.0)
<12 months ago	59.4 (58.2-60.5)	2.1 (1.8-2.5)	12.1 (11.3-13.0)	56.4 (55.1-57.6)	1.6 (1.2-1.9)	10.6 (9.8-11.5)
Dental insurance						
No	54.4 (52.9-56.0)	9.4 (8.7-10.2)	22.6 (21.4-23.9)	48.9 (47.2-50.5)	6.5 (5.8-7.2)	20.0 (18.7-21.2)
Yes	45.6 (44.0-47.1)	3.1 (2.6-3.5)	11.0 (10.1-11.9)	51.1 (49.5-52.8)	1.7 (1.4-2.0)	8.3 (7.5-9.0)

Amaranth: statistical difference between groups only at each time point.

Blue: statistically significant difference between groups only at 2 time points.

Green: statistically significant difference not only between groups at each time point, but also between groups at 2 time points.

⁎ CALD defined by country of birth not being Australia AND English not the primary language spoken at home.

Over the 14-year survey period, the prevalence of edentulism declined from 6.4% to 4.0%, and non-functional dentition from 17.1% to 13.8%. Notable reductions in edentulism were observed among those aged 55+ (19.6%-11.1%), females (7.6%-4.7%), males (5.2%-3.4%), residents in both regional/remote (9.2%-5.4%) and major cities (5.1%-3.5%), and in the lowest (17.1%-7.2%), second lowest (8.7%-5.8%), and highest (1.4%-0.7%) income groups. Similar trends were seen among those whose last dental visit was over 12 months ago (12.7%-7.1%) and those with (3.1%-1.7%) or without (9.4%-6.5%) dental insurance. For non-functional dentition, decreases were noted among those aged 35-54 (8.4%-5.9%) and 55+ (47.1%-35.5%), residents in regional/remote (21.9%-18.2%) and major cities (14.7%-12.1%), the lowest income group (38.6%-23.6%), those with last dental visit >12 months ago (43.6%-17.8%), and those with (11.0%-8.3%) or without (22.6%-20.0%) dental insurance (Table 1).

**Fig. 1:**
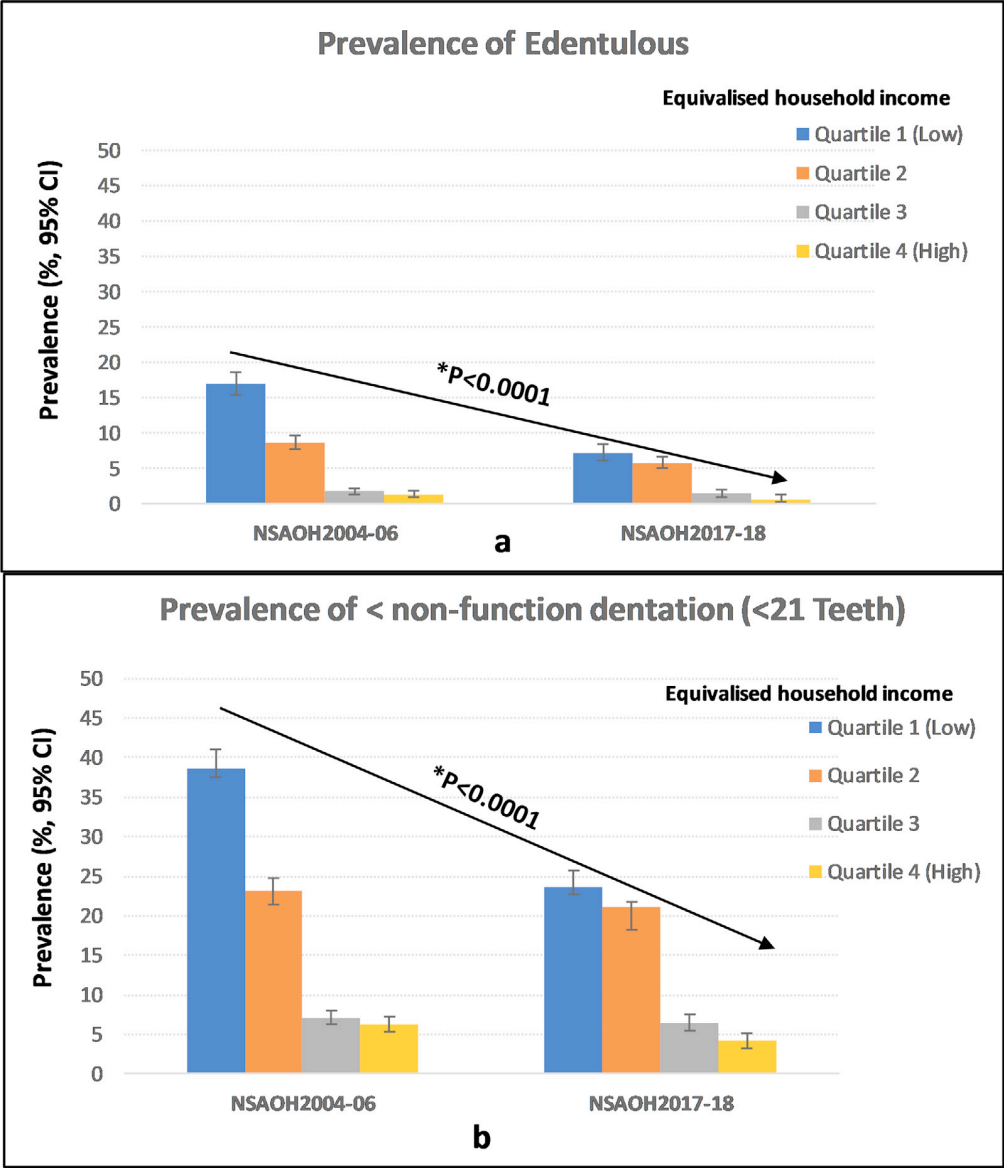
Prevalence of edentulous (a) and non-functional dentition (<21 teeth) (b) by equivalized household income (*the Cochran-Armitage test).

Figure 1 presents the prevalence of edentulism (‘a’) and nonfunctional dentition (‘b’) across all household income quartiles and the 2 time points. With the increase of household income from the lowest (Q1) to the highest (Q4), the prevalence of edentulism and non-functional dentition showed a clear downward trend was observed in both NSAOH-1 and NSAOH-2, with p-values < .0001 in each case.

Across all household income quartiles, both unadjusted and adjusted prevalence of edentulism was lower in NSAOH-2 compared to NSAOH-1 (Table 2). The most notable reduction was observed in the lowest income group (Q1), where prevalence declined from 17.1% to 7.2% (PD = 9.9). This was followed by the second lowest income group (Q2), with a reduction from 8.7% to 5.8% (PD = 2.9), and the highest income group (Q4), from 1.4% to 0.7% (PD = 0.7). A similar trend was observed after adjusting for age and sex. When comparing the lowest to the highest income quartile, the adjusted prevalence difference was 14.1 percentage points in NSAOH-1 (15.3% vs. 1.2%) and 5.9 in NSAOH-2 (6.5% vs. 0.6%). These findings indicate a narrowing of socioeconomic inequality in edentulism over the 14 years (PDs = 8.2). Similar patterns were also observed in the sensitivity analysis, as shown in Table 2.

**Table 2:** Unadjusted and adjusted prevalence and prevalence difference (PD) for edentulism, by household income status.

Original data	Unadjusted		Adjusted*	
	Prevalence (95% CI)	PD (95% CI)	Prevalence (95% CI)	PD (95% CI)
Household income Q1 (lowest)				
NSAOH1	**17.1 (15.4-18.7)**	ref	**15.3 (13.7-16.9)**	ref
NSAOH2	**7.2 (6.1-8.4)**	9.9 (9.3-10.3)	**6.5 (5.4-7.5)**	8.8 (8.3-9.4)
Household income Q2				
NSAOH1	**8.7 (7.7-9.7)**	ref	**7.6 (6.8-8.5)**	ref
NSAOH2	**5.8 (5.0-6.6)**	2.9 (2.7-3.1)	**5.1 (4.4-5.8)**	2.5 (2.4-2.7)
Household income Q3				
NSAOH1	1.8 (1.4-2.2)	ref	1.6 (1.2-1.9)	ref
NSAOH2	1.5 (0.9-2.1)	0.3 (0.1-0.5)	1.3 (0.8-1.8)	0.3 (0.1-0.4)
Household income Q4 (highest)				
NSAOH1	1.4 (1.0-1.8)	ref	1.2 (0.8-1.6)	ref
NSAOH2	0.7 (0.3-1.0)	0.7 (0.6-0.8)	0.6 (0.3-0.9)	0.6 (0.5-0.7)

	Sensitivity analysis			
Imputed data	Unadjusted		Adjusted*	
	Prevalence (95% CI)	PD (95% CI)	Prevalence (95% CI)	PD (95% CI)

Household income Q1 (lowest)				
NSAOH1	**15.8 (14.3-17.4)**	ref	**14.1 (12.7-15.6)**	ref
NSAOH2	**7.7 (6.7-8.8)**	8.1 (7.6-8.6)	**6.9 (5.9-7.8)**	7.2 (6.8-7.8)
Household income Q2				
NSAOH1	**8.1 (7.2-9.0)**	ref	**7.1 (6.3-7.9)**	ref
NSAOH2	**4.9 (4.3-5.6)**	3.2 (2.9-3.4)	**4.3 (3.7-4.9)**	2.8 (2.6-3.0)
Household income Q3				
NSAOH1	1.9 (1.5-2.3)	ref	1.6 (1.3-2.0)	ref
NSAOH2	1.5 (1.0-2.0)	0.4 (0.3-0.5)	1.3 (0.9-1.7)	0.3 (0.2-0.4)
Household income Q4 (highest)				
NSAOH1	**1.4 (1.0-1.8)**	ref	**1.2 (0.9-1.6)**	ref
NSAOH2	**0.6 (0.4-0.9)**	0.8 (0.6-0.9)	**0.6 (0.3-0.8)**	0.6 (0.5-0.8)

⁎ Adjusted for age and sex. The difference is statistically significant as denoted by non-overlapping 95% confidence intervals (Highlighted in black bold).

Across all household income quartiles, both unadjusted and adjusted prevalence of non-functional dentition was lower in NSAOH-2 than in NSAOH-1 (Table 3). The most notable decrease was observed in the lowest household income group (Q1), which declined from 38.6 to 23.6 (PD = 15.0), followed by the highest income group (Q4), which decreased from 6.3 to 4.2 (PD = 2.1). After adjusting for age and sex, this pattern remained consistent. When comparing the lowest and highest income quartiles, the prevalence difference was 29.4 in NSAOH-1 (35.0 vs. 5.6) and 17.5 in NSAOH-2 (21.3 vs. 3.8). These findings indicate a reduction in social inequality in the prevalence of non-functional dentition over time (PD = 11.9). Consistent findings were observed in the sensitivity analysis (Table 3), further supporting the robustness of the results.

**Table 3:** Unadjusted and adjusted prevalence and prevalence difference (PD) for non-functional dentition, by household income status.

Original data	Unadjusted		Adjusted*	
	Prevalence (95% CI)	PD (95% CI)	Prevalence (95% CI)	PD (95% CI)
Household income Q1 (low)				
NSAOH1	**38.6 (36.2-41.0)**	ref	**35.0 (32.7-37.3)**	ref
NSAOH2	**23.6 (21.5-25.7)**	15.0 (14.7-15.3)	**21.3 (19.3-23.3)**	13.7 (13.4-14.0)
Household income Q2				
NSAOH1	23.1 (21.4-24.7)	ref	20.6 (19.1-22.1)	ref
NSAOH2	21.1 (18.3-21.8)	2.0 (1.2-2.9)	17.8 (16.2-19.5)	2.8 (2.6-4.4)
Household income Q3				
NSAOH1	7.1 (6.2-8.1)	ref	6.4 (5.5-6.7)	ref
NSAOH2	6.5 (5.5-7.5)	0.6 (0.5-0.7)	5.8 (4.8-6.7)	0.6 (0.1-0.7)
Household income Q4 (high)				
NSAOH1	**6.3 (5.3-7.3)**	ref	**5.6 (4.7-6.5)**	ref
NSAOH2	**4.2 (3.3-5.1)**	2.1 (2.0-2.2)	**3.8 (3.0-4.6)**	1.8 (1.7-1.9)

	Sensitivity analysis			
Imputed data	Unadjusted		Adjusted*	
	Prevalence (95% CI)	PD (95% CI)	Prevalence (95% CI)	PD (95% CI)

Household income Q1 (low)				
NSAOH1	**36.3 (34.0-38.5)**	ref	**32.7 (30.5-35.0)**	ref
NSAOH2	**23.9 (22.0-25.8)**	12.4 (12.0-12.7)	**21.5 (19.7-23.3)**	11.2 (10.8-11.7)
Household income Q2				
NSAOH1	**22.1 (20.6-23.5)**	ref	**19.7 (18.3-21.1)**	ref
NSAOH2	**17.1 (15.6-18.5)**	5.0 (4.9-5.9)	**15.0 (13.7-16.4)**	4.7 (4.6-4.9)
Household income Q3				
NSAOH1	7.1 (6.2-8.0)	ref	6.3 (5.5-7.1)	ref
NSAOH2	6.7 (5.8-7.6)	0.4 (0.4-0.5)	5.9 (5.1-6.8)	0.4 (0.3-0.6)
Household income Q4 (high)				
NSAOH1	**6.0 (5.0-6.9)**	ref	**5.3 (4.5-6.1)**	ref
NSAOH2	**4.1 (3.4-4.9)**	1.9 (1.6-2.0)	**3.7 (3.0-4.4)**	1.6 (1.5-1.8)

⁎ Adjusted for age and sex. The difference is statistically significant as denoted by non-overlapping 95% confidence intervals (Highlighted in black bold).

Table 4 presents the multivariable analyses comparing the 2 surveys and examining the explanatory indicators associated with the prevalence of edentulism and nonfunctional dentition among Australian adults. Compared to NSAOH-2, participants in NSAOH-1 had a 1.7 times higher prevalence of edentulism (OR = 1.72, 95% CI: 1.56-1.90) and a 1.3 times higher prevalence of non-functional dentition (OR = 1.34, 95% CI: 1.27-1.42). A higher prevalence of edentulism and non-functional dentition was also observed among individuals residing in remote areas, those with lower household income (Q1-Q3), those whose last dental visit was more than 12 months ago, and those without dental insurance. Lower prevalence of edentulism and non-functional dentition was observed among younger individuals (under 55 years) and those identifying as CALD. Males also exhibited a lower prevalence of edentulism compared to their female counterparts.

**Table 4:** Multivariable association between tooth loss and surveys and explanatory variables (OR, 95% CI).

	Edentulism	Non-functional dentition
	OR (95% CI)	OR (95% CI)
Surveys		
NSAOH 1	1.72 (1.56-1.90)⁎⁎⁎	1.34 (1.27-1.42)⁎⁎⁎
NSAOH 2	ref	ref
Age group (years)		
15-34	0.00 (0.00-0.01)⁎⁎⁎	0.02 (0.02-0.03)⁎⁎⁎
35-54	0.13 (0.11-0.16)⁎⁎⁎	0.21 (0.19-0.23)⁎⁎⁎
55+	ref	ref
Sex		
Male	0.76 (0.69-0.84)⁎⁎⁎	0.98 (0.92-1.03)
Female	ref	ref
Residential location		
Regional/remote	1.18 (1.08-1.30)⁎⁎	1.13 (1.06-1.19)⁎⁎⁎
Major city	ref	ref
CALD status*		
CALD	0.74 (0.60-0.91)⁎⁎	0.89 (0.79-0.99)*
All others	ref	ref
Equivalised household income		
Quartile 1 (lowest)	3.05 (2.43-3.84)⁎⁎⁎	2.61 (2.33-2.93)⁎⁎⁎
Quartile 2	2.67 (2.13-3.35)⁎⁎⁎	2.30 (2.06-2.58)⁎⁎⁎
Quartile 3	1.46 (1.12-1.90)⁎⁎	1.36 (1.20-1.55)⁎⁎⁎
Quartile 4 (highest)	ref	ref
Last dental visit		
12+ months ago	4.40 (3.90-4.96)⁎⁎⁎	1.52 (1.43-1.61)⁎⁎⁎
<12 months ago	ref	ref
Dental insurance		
No	1.59 (1.42-1.79)⁎⁎⁎	1.47 (1.38-1.57)⁎⁎⁎
Yes	ref	ref

OR, odds ratio.

⁎⁎⁎ p-value < .0001.

⁎⁎ p-value < .001.

⁎ *P* < .05.

## Discussion

Our study found social inequalities in tooth loss, both edentulism and non-functional dentition, among Australian adults from 2004-06 to 2017-18. The inequalities were observed between household groups at each time point, with a decrease of household income from the highest (Q4) to the lowest (Q1) equating to an increase in the prevalence of edentulism and non-functional dentition. Ultimately, however, the inequalities decreased across 14 years. Age, sex, place of residence, and dental health-related behaviours (irregular dental visit and without dental insurance) were important factors affecting the equalities.

Our study provided some evidence that socioeconomic status determines oral health: low household income has been associated with a higher risk of tooth loss, likely due to limited financial resources for dental care and preventive measures. Individuals with lower incomes may also face additional barriers to accessing dental services, such as lack of transportation, flexible work schedules or dental insurance. As a result, they may be less likely to attend regular dental visits. Evidence suggests^17^ that socioeconomic status significantly influences access to dental insurance, contributing to a pro-wealth disparity. Individuals without dental insurance are more likely to delay or have irregular dental visits,^18^ which can lead to poor oral health and increased risk of tooth loss. Additionally, people living in remote or very remote areas face not only challenges in accessing dental services but also reduced quality of care due to a shortage of dental professionals.^19^^,^^20^ As a result, oral health is not guaranteed, leading to lose teeth and loss of teeth, seriously affecting the quality of life.^21^

We observed that the prevalence differences (PDs) for income Q3 are lower than those in the other quartiles. Several factors may explain this pattern: (1) Threshold effects—individuals in Q3 may fall between the extremes, above the level of critical deprivation but below the point where high-resource advantages become evident, resulting in smaller PDs; (2) Heterogeneity within the quartile—Q3 may encompass a diverse group with varying educational backgrounds and health behaviours, such as smoking, diet, and oral hygiene, which could dilute overall associations; and (3) Nonlinear improvements in access to dental care—increases in income may not translate proportionally to better healthcare access, particularly in the presence of structural barriers like limited dental insurance or inadequate transportation.

The social inequities in the prevalence of tooth loss (both edentulism and non-functional dentition) decreased between the most socially disadvantaged and advantaged across 2 time points. Previous multinational studies^1^^,^^22^ found that Australia exhibited lower absolute socioeconomic inequalities in tooth loss compared to several other OECD countries, including the United States, Brazil and Chile. This result is consistent with a previous study^23^ and suggests that the provision of dental services in Australia is increasing, driven primarily by urban areas rather than rural ones, and that perhaps more people can afford and can access dental care, and possibly that individual oral health awareness has improved. It is undeniable that community water fluoridation reduces tooth decay, which is a leading cause of tooth loss.^24^ However, the prevalence of tooth loss in the most socially disadvantaged group was much higher than in the most advantaged group in 2017-18, because most dental care in Australia is privately funded, a 2-tier system has emerged in which wealthier individuals receive timely and comprehensive services, while low-income populations are left to depend on under-resourced public clinics or forgo care altogether,^25^ which suggests that the benefits of oral health service provision, such as Medicare coverage for dental care, still require significant improvement, particularly for socially disadvantaged populations. The Australian Government is developing the National Oral Health Plan 2025–2034, aiming to improve oral health outcomes and reduce inequalities.^26^ Enhancing dental health literacy at the population level remains essential for increasing awareness of disease prevention.^27^

Strengths of the study include assessing and comparing 2 large, representative samples of Australian adults and reducing the need for clinical oral examinations. This can significantly lower costs, as these assessments typically require trained dental professionals, specialized equipment, and logistical support such as travel or infrastructure. In contrast, self-reported oral health measures can be obtained at a much lower cost through questionnaires or interviews.^28^ This study has several limitations: (1) the use of cross-sectional data limits the ability to assess the temporality and direction of relationships between variables, (2) missing data on household income (7.5%) may introduce bias. However, the use of weighted data and a large sample size helps to mitigate random error, and (3) the study did not assess income-related relative inequalities in tooth loss, which may result in inaccurate or imprecise estimates.

Although we observed that the association between income and tooth loss attenuated after adjusting for age, sex, and other variables, suggesting that these factors may lie on the causal pathway, we did not perform a formal mediation analysis. Our study was not designed within a causal inference framework, and we did not apply specific methods to decompose total effects into direct and indirect (mediated) effects. As such, we cannot draw conclusions about mediation. Future research using causal mediation approaches would be valuable to better elucidate whether and how factors such as dental service use or insurance coverage mediate the relationship between income and tooth loss.

## Conclusion

Our study indicated social inequalities in both edentulism and non-functional dentition among Australian adults across a 14-year time period. Age, sex, place of residence, irregular dental visits, and lack of dental insurance were important factors associated with tooth loss.

Addressing social inequality in tooth loss requires a multifaceted approach. It necessitates improving access to dental care for individuals with lower SES, promoting regular dental visits and preventive care, and addressing the underlying lifestyle factors that contribute to tooth loss. By understanding and addressing these inequalities, we can work towards achieving a more equitable oral health system that benefits all members of society.

## Declaration of competing interest

The authors declare that they have no known competing financial interests or personal relationships that could have appeared to influence the work reported in this paper.
